# Persistent Homology Metrics Reveal Quantum Fluctuations and Reactive Atoms in Path Integral Dynamics

**DOI:** 10.3389/fchem.2021.624937

**Published:** 2021-03-05

**Authors:** Yunfeng Hu, Phonemany Ounkham, Ondrej Marsalek, Thomas E. Markland, Bala Krishmoorthy, Aurora E. Clark

**Affiliations:** ^1^ Department of Mathematics and Statistics, Washington State University, Pullman, WA, United States; ^2^ Department of Chemistry, Washington State University, Pullman, WA, United States; ^3^ Faculty of Mathematics and Physics, Charles University, Prague, Czech; ^4^ Department of Chemistry, Stanford University, Stanford, CA, United States; ^5^ Department of Mathematics and Statistics, Washington State University, Vancouver, WA, United States

**Keywords:** path integral molecular dynamics, persistent homology, quantum delocalization, proton transfer, Wasserstein distances

## Abstract

Nuclear quantum effects (NQEs) are known to impact a number of features associated with chemical reactivity and physicochemical properties, particularly for light atoms and at low temperatures. In the imaginary time path integral formalism, each atom is mapped onto a “ring polymer” whose spread is related to the quantum mechanical uncertainty in the particle’s position, i.e., its thermal wavelength. A number of metrics have previously been used to investigate and characterize this spread and explain effects arising from quantum delocalization, zero-point energy, and tunneling. Many of these shape metrics consider just the instantaneous structure of the ring polymers. However, given the significant interest in methods such as centroid molecular dynamics and ring polymer molecular dynamics that link the molecular dynamics of these ring polymers to real time properties, there exists significant opportunity to exploit metrics that also allow for the study of the fluctuations of the atom delocalization in time. Here we consider the ring polymer delocalization from the perspective of computational topology, specifically persistent homology, which describes the 3-dimensional arrangement of point cloud data, (i.e. atomic positions). We employ the Betti sequence probability distribution to define the ensemble of shapes adopted by the ring polymer. The Wasserstein distances of Betti sequences adjacent in time are used to characterize fluctuations in shape, where the Fourier transform and associated principal components provides added information differentiating atoms with different NQEs based on their dynamic properties. We demonstrate this methodology on two representative systems, a glassy system consisting of two atom types with dramatically different de Broglie thermal wavelengths, and ab initio molecular dynamics simulation of an aqueous 4 M HCl solution where the H-atoms are differentiated based on their participation in proton transfer reactions.

## 1 Introduction

In recent years, path integral (PI) methods have seen significant application as a means to study nuclear quantum effects (NQEs), such as those arising from zero-point energy and tunneling, in chemical systems. In the imaginary time PI approach, each atom is described as a ring polymer composed of a set of beads where the adjacent beads interact via harmonic springs (Feynman and Hibbs, 1963; Chandler and Wolynes, 1981; Parrinello and Rahman, 1984). As the mass of the nuclei or the temperature of the system increases, the stiffness of the harmonic spring between the beads is increased, the polymer shrinks, and the ring polymer representation of the atom becomes more “localized”. Conversely, for lower temperatures or for lighter particles, the weaker coupling between the beads allows the ring polymer to adopt a range of shapes reflecting the quantum mechanical delocalization in the atom’s position. The quantum mechanical uncertainty in the atom’s position is composed of the distribution of the centroid position and the ring polymer’s spread.

NQEs have been demonstrated to affect hydrogen bond strengths, and thus the physicochemical, structural, and dynamic properties of protic solvents like water (Hardy et al., 2001; Morrone and Car, 2008; Habershon et al., 2009; Paesani and Voth, 2009; Pamuk et al., 2012; Harada et al., 2013; Ceriotti et al., 2016; Kim et al., 2017; Ruiz Pestana et al., 2018). The structure and dynamics of the species within acidic media has also received significant attention. For example, NQEs are observed to increase delocalization within protonated structures and as such enhance proton transfer within acidic systems (Ivanov et al., 2015; Marsalek and Markland, 2017; Napoli et al., 2018; Kawashima et al., 2018).

Several metrics have been proposed to characterize atomic delocalization in path integral systems. The imaginary-time mean square displacement (Berne and Thirumalai, 1986) evaluates a correlation function along the ring polymer. A set of shape metrics have also been introduced that characterize the anisotropy of the ring polymer in different chemical environments. The extension of the ring polymer is projected along a particular coordinate of interest e.g., in the case the proton transfer between two oxygen atoms projecting along the O-O coordinate (Benoit and Marx, 2005; Schran et al., 2018). By constructing idealized ellipsoid models of the bead density and their associated principal axes, an approximate shape of the distribution can be obtained (cigar-like or disk-like). Complementary, is the construction and analysis of the radius of gyration (R_*g*_) of the ring polymer, defined as the average root mean squared distance of the replicas from the polymer center (or centroid), or related quantities such as the ratio of R_*g*_ values for different atoms, and gyration tensors (Markland et al., 2011, 2012; Dreschel-Grau and Marx, 2014; Schran et al., 2018). These shape metrics thus provide a route to analyze NQEs once a relevant atom has been identified. However, it leaves open the possibility to investigate a broader set of shape metrics to capture the changes in the *global* shape of the ring polymer and thus identify *a priori* atoms undergoing interesting changes in their “quantumness”. In particular, these methods only utilize the static information obtained from a path integral molecular dynamics (PIMD) or path integral Monte Carlo (PIMC) sampling. While originally the dynamics obtained by PIMD was introduced purely as a tool to sample the quantum ensemble (Parrinello and Rahman, 1984; Tuckerman et al., 1993), methods such as centroid molecular dynamics (Cao and Voth, 1994; Jang and Voth, 1999) (CMD) and ring polymer molecular dynamics (Craig and Manolopoulos, 2004; Habershon et al., 2013) (RPMD) have demonstrated that for systems where the quantum coherence of the nuclei is rapidly damped that classical evolution under the imaginary time ring polymer Hamiltonian can be used to predict the dynamics of a quantum system. This opens the door to using the specific time series information of the global ring polymer configurations generated by these methods to identify quantum events.

Within the last decade, concepts from the mathematical field of algebraic topology have been combined with computational methods to characterize the global shape of data (Carlsson, 2009). Termed computational topology or topological data analysis (TDA), this field has seen rapid developments (Edelsbrunner and Harer, 2009). Persistent homology is a TDA method that produces compact summaries of the global shape and topology of sets of points in the form of barcodes (Ghrist, 2008). Given a collection of data sets (ring polymers representing atoms in our case), persistent homology provides an objective way to quantify and compare global shapes of the data sets by measuring distances between their barcodes. Statistical analyses on collections of such barcode distances may also be used to distinguish between different distributions. Here we apply persistent homology to study the time-dependent fluctuations of the ring polymers arising from RPMD and thermostatted RPMD (TRPMD) (Rossi et al., 2014) simulations and assess its ability to detect chemically meaningful information about NQEs.

In particular, we compare and contrast different shape and persistent homology metrics for two different chemical systems. The first system is a Kob–Andersen glass that contains two atom types of dramatically different quantum mechanical uncertainty. Not only is persistent homology able to elucidate variations in ring-polymer shape, but the Wasserstein distance between adjacent snapshots in time (which measures the change in the shape of the ring polymer), and its associated Fourier transform are found to be remarkably different for the two different atom types. In the second system, we examine the ability of the shape and persistent homology metrics to identify proton-transferring (PT) vs. non-PT H-atoms in an *ab initio* path integral simulation of an aqueous 4 M HCl solution. Again, a pronounced difference is observed in the Fourier transform of the Wasserstein distance, where PT H-atoms have significantly more fluctuation in shape than their non-PT counterparts. This observation paves the way for employing persistent homology in the study of a wide variety of chemical systems where NQEs are relevant, to not only identify atoms that have different nuclear behavior, but understand the change in quantum delocalization of an atom over time and along complex reaction coordinates.

## 2 Computational Methods

### 2.1 Static Atomic Uncertainty Metrics

In this work we consider several metrics that reflect the distribution of the distances of replicas relative to the centroid of the ring polymer as well as between the replicas themselves. Results for these quantities are included in the Supplementary Information for comparison and completeness. For a system of *p* replicas of $N_{A}$ atoms, we denote the position of replica *k* of atom *j* as ${\text{r}}_{j}^{\left(k\right)}$ and the position of the centroid of atom *j* as ${\bar{\text{r}}}_{j}$. The gyration radius of atom *j* for any given configuration of the ring polymer is defined as the root mean square of the distance between the centroid and the replicas,

$$R_{g,j}=\sqrt{\frac{1}{P}\sum_{k=1}^{P}{\left|{\text{r}}_{j}^{\left(k\right)}-\bar{{\text{r}}_{j}}\right|}^{2}}.$$ (1)

This quantity is then most commonly averaged over all equivalent atoms and over the ensemble sampled by the simulation. More generally, we can examine the gyration tensor of atom *j* defined as

$$S_{\left(j\right)}=\frac{1}{P}\sum_{k=1}^{P}\left({\text{r}}_{j}^{\left(k\right)}-\bar{{\text{r}}_{j}}\right)\left({\text{r}}_{j}^{\left(k\right)}-\bar{{\text{r}}_{j}}\right).$$ (2)

Note that the subscript (j) of the tensor specifies index of the atom, and not its rank. The tensor can be represented by a symmetric 3×3 matrix whose off-diagonal entries give the xy,yz, and xz components of the tensor. Diagonalization of the 3×3 matrix representing the gyration tensor yields eigenvectors that describe the principal directions of the distribution of points and eigenvalues that describe the spread of the distribution in these directions. If we denote the ordered eigenvalues $\lambda_{x}^{2}$, $\lambda_{y}^{2}$, and $\lambda_{z}^{2}$, they can be used to obtain the gyration radius as $R_{g,j}^{2}=\lambda_{x}^{2}+\lambda_{y}^{2}+\lambda_{z}^{2}$ and additional shape descriptors common in polymer and macromolecular science as follows (Mattice and Suter, 1994). The asphericity

$$b=\frac{3}{2}\lambda_{z}^{2}-\frac{R_{g}^{2}}{2},$$ (3)

describes the deviation from a fully symmetric distribution (for which b=0), whereas the acylindricity

$$c=\lambda_{y}^{2}-\lambda_{x}^{2},$$ (4)

emphasizes symmetry about any two coordinate axes. Relative shape anisotropy is defined as

$$\kappa^{2}=\frac{3}{2}\frac{\lambda_{x}^{4}+\lambda_{y}^{4}+\lambda_{z}^{4}}{{\left(\lambda_{x}^{2}+\lambda_{y}^{2}+\lambda_{z}^{2}\right)}^{2}}-\frac{1}{2},$$ (5)

where a value of zero only occurs when all points are spherically symmetric, while a value of one is observed if all points are on a line. Metrics based on the inter-bead distances could include the distribution of all centroid to bead distances, or the distribution of pairwise distances between individual beads within the ring polymer, $\left\{\left|r_{j}^{\left(k\right)}-r_{j}^{\left(l\right)}\right|:1\leq k\leq l\leq P\right\}$. The imaginary time mean-square displacement (iMSD) is also frequently employed to study a variety of aspects of path-integral simulations. Variations in the iMSD are characteristic of the spread of the ring polymer and also provide information about short-time dynamics (Tuckerman, 2010). It is calculated as

$$\Delta r^{2}\left(i\tau \right)=\langle \frac{1}{N_{A}P}\sum_{j=1}^{N}\sum_{k=1}^{P}{\left|{\text{r}}_{j}^{\left(k\right)}-{\text{r}}_{j}^{\left(k+l\right)}\right|}^{2}\rangle ,$$ (6)

where angle brackets denote averaging over the sampled ensemble, τ=lβℏ/P and replica indices should be taken modulo *p* in the ring polymer.

### 2.2 Dynamic Shape Metrics From Persistent Homology

As an extension of the methods provided above, it is intriguing to combine the information contained within shape metrics of the polymer with its dynamic behavior. Toward this end we consider homology, the method from classical algebraic topology that captures how a space is connected. Herein, we first describe the general principles of homology and persistent homology, as well as known distance metrics to measure changes in topological features, as they are both applied to the ring polymer dynamics trajectories.

#### 2.2.1 Persistent Homology as Metric of Shape

In the setting of homology directly amenable to computation, the space is modeled as a combinatorial object called the simplicial complex, which is a collection of vertices, edges, triangles, and higher order simplices glued together “nicely” (Munkres, 1984). For instance, a triangular mesh is a 2-dimensional simplicial complex. The ranks of the homology groups, termed *Betti numbers* and denoted by $\beta_{i}$ in dimension *i*, have intuitive interpretations for small dimensions. In particular, $\beta_{0}$ counts the number of connected components in the object or space, $\beta_{1}$ counts the number of loops or holes, and $\beta_{2}$ counts the number of enclosed voids. Since we are interested in the global shapes of ring polymers that naturally form loops, we study the first Betti number $\beta_{1}$.

Persistent homology (Edelsbrunner et al., 2002) produces a more comprehensive picture (than simple homology) of the shape of space by constructing a sequence of growing simplicial complexes, rather than a single complex. Changes in $\beta_{i}$ values are tracked across this sequence, and this information is presented in a compact form as a *barcode* (one barcode in each dimension *i*). Such persistent homology representations come with stability guarantees—small changes in input produce only small changes in the representation (Cohen-Steiner et al., 2007).

**FIGURE 1:**
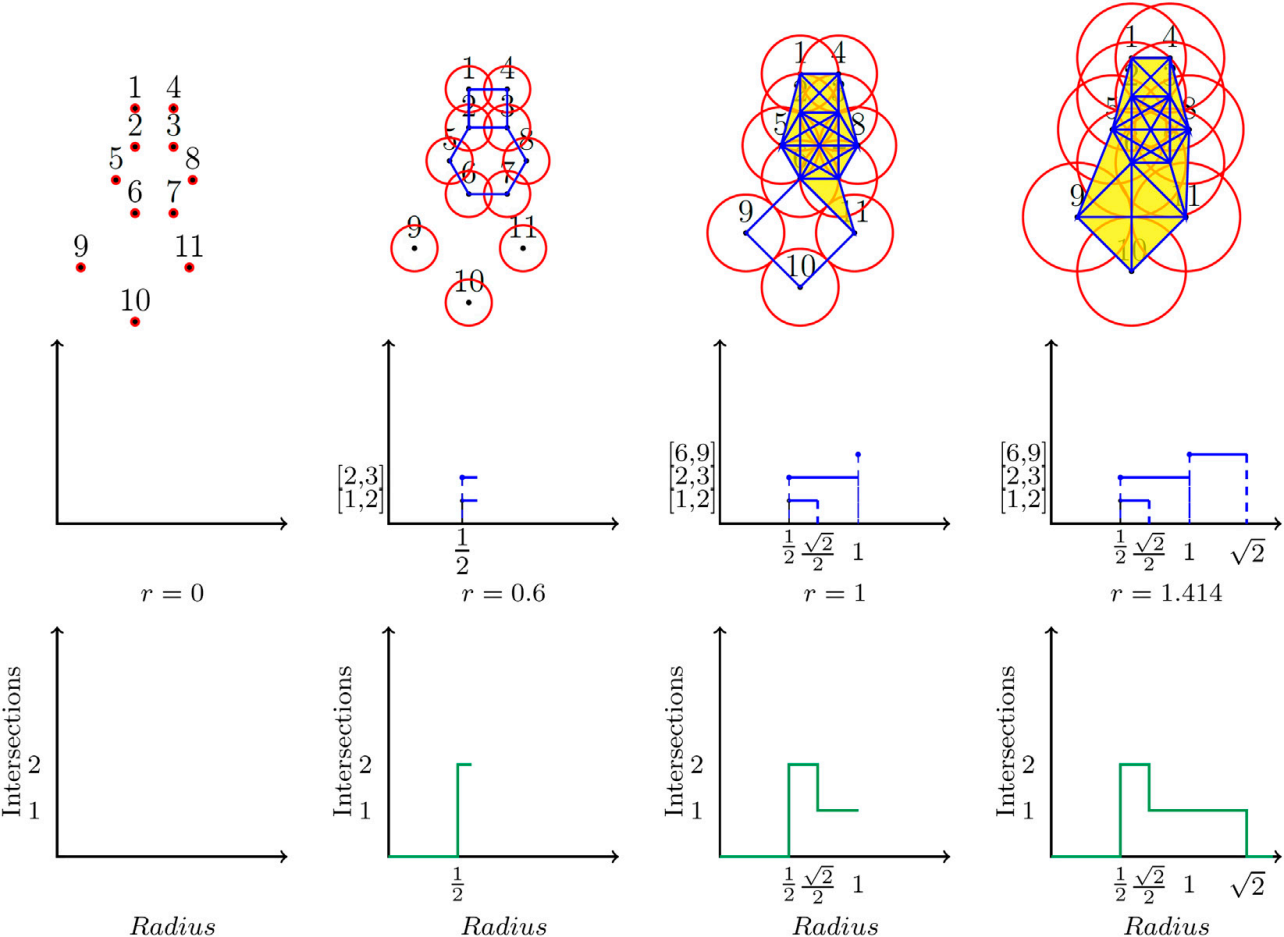
Top Row: A ball of growing radius *r* (using the units of the coordinate system) is centered at each bead. Middle Row: A $\beta_{1}$ barcode records the birth and death of the holes. Each hole is “represented” by one of its edges, which is listed on the vertical axis ([1,2],[2,3],[6,9]). Bottom Row: A Betti sequence is constructed by sliding a vertical line at each radius and keeping track of the numbers of intersection of the line and the barcode.

Given the collection of beads in a ring polymer, we consider a ball of radius *r* centered at each bead (Figure 1). We systematically grow the radius *r* from 0 to infinity (in this study, we measure *r* in Angstroms). Observe that as the radius grows, balls centered at beads that are close to each other will intersect before those centered at beads that are farther apart. The intersections of these balls over the entire range of values of *r* capture all information about the global shape of the ring polymer. These intersections are used to define the Vietoris-Rips (VR) complex (Edelsbrunner and Harer, 2009) of the ring polymer. When r=0, the VR complex consists of the individual points associated with the beads of the ring polymer, as the balls have no intersections. As *r* is increased, the intersection of a pair of balls is captured by adding the edge connecting the points to the VR complex. Triangles, tetrahedra, and higher order simplices are added to the VR complex to capture higher order intersections of balls. As the VR complex grows, small connected components merge into bigger connected components, and holes as well as voids appear and disappear.

The top row in Figure 1 displays the construction of a VR complex for a ring polymer with 11 beads. 2D balls centered at the points (representing the beads) are shown as circles. At r=0 (first figure on the left), none of the balls intersect, and hence the VR complex consists of individual points representing the beads. At $r=\frac{1}{2}$, edges added to capture pairwise intersections of the balls form two loops in the VR complex, shown as a square and a hexagon in the second figure. At $r=\frac{\sqrt{2}}{2}$, balls centered at beads one to four intersect pairwise, and hence simplices (tetrahedron 1,234, and its component triangles) are added to the VR complex to fill up the square loop, thus “killing” this feature of the topology. The barcode in the second row of Figure 1 records the birth and death of each loop in the VR complex as the radius is increased. The hexagonal loop formed by beads 3, 2, 5, 6, 7, and 8, for instance, is born, (i.e. formed) at $r=\frac{1}{2}$, and dies, (i.e. is closed up) at r=1. The complete $\beta_{1}$ barcode is shown in the fourth (last) figure, with all holes closed up at $r=\sqrt{2}$. We can use this barcode as the representation of the shape of the ring polymer.

#### 2.2.2 Fluctuations in Shape

We could compare the shapes of two ring polymers by comparing their $\beta_{1}$ persistence barcodes. To quantify this comparison, we want to compute a distance between the barcodes. We are using the word *distance* in the mathematical sense: a distance is a function that accepts two distributions as input, and returns a nonnegative real number which measures how close the two distributions are. To this end, we want to convert each barcode to a vector with the same number of entries, and then compute the distance between the corresponding vectors. We build a *Betti sequence* by sliding a vertical line across each radius value and keeping track of the intersection of the line and the barcode (bottom row, Figure 1). For example, a vertical line at $r=\frac{1}{2}$ intersects the barcode twice as there are 2 bars at $r=\frac{1}{2}$. Thus the Betti sequence is constructed by recording the number of bars at each radius. Hence, denoting h(r) to be the number of intersections at radius *r*, we define the Betti sequence as ${\left\{h\left(r\right)\right\}}_{r\in \left[0,1\right]}$. The choice of upper bound of r=1 is motivated by the observation that for all ring polymers we considered in this study, the holes were closed well before the radius value of r=1 in each chemical system (*r* = 1 Bohr for the Kob–Andersen glass and *r* = 1 Å for the proton transfer example). In other words, h(r)=0 for r≥1. The resolution at which we cover [0,1] is guided by the barcodes—we increment *r* in steps fine enough to distinguish births and deaths of each bar. We used 400 steps for the Kob–Anderson glass system and 1,000 steps for the second system studying proton transfer.

In the final step, we normalize this Betti sequence to create the *Betti sequence probability distribution* indexed by the number of intersections. We use the maximum number of intersections observed in any ring polymer as the common number of intersections used in all cases, thus standardizing the lengths of all Betti sequence probability distributions. Each such distribution adds up to a total probability of 1, by definition. Note that there are other vectorizations of persistence barcodes or diagrams known, e.g., persistence landscapes (Bubenik, 2015) and persistence images (Adams et al., 2017). These constructions are arguably more general than our Betti sequences. At the same time, we found the Betti sequences simpler to compute, and they served our purpose in this study of ring polymers efficiently.

**FIGURE 2:**
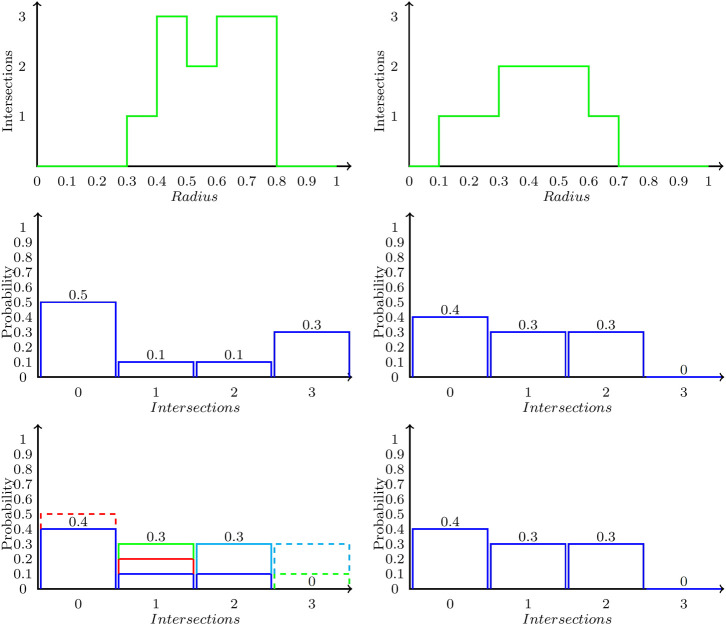
Illustration of Wasserstein distance computation between two Betti sequence probability distributions. Top row shows two Betti sequences. The second row shows the corresponding Betti sequence probability distributions P **(left)** and Q **(right)**. The last row shows the optimal transformation of P into Q, which consists of three steps: moving the red box from bin 0 to one contributing 0.1×|0−1|=0.1, the green box from bin three to one contributing 0.1×|1−3|=0.2, and the cyan box from bin three to two contributing 0.2×|2−3|=0.2. Therefore, the Wasserstein distance is 0.1+0.2+0.2=0.5.

Comparison of two different ring polymer shapes can be made by calculating the Wasserstein distance (WD) between the Betti sequence probability distributions. Stability results have recently been presented for WD of persistent barcodes (Skraba and Turner, 2020). The Wasserstein distance (Villani, 2009), also termed the *Earth Mover’s Distance* (Rubner et al., 2000) is a metric that measures the distance between the two normalized distributions as the cost of transforming one into another. We present the definition and then illustrate steps in the WD computation using Figure 2. More generally, let $K={\left\{K\left(i\right)\right\}}_{i=1}^{p}$ and $L={\left\{L\left(j\right)\right\}}_{j=1}^{q}$ be two normalized probability distributions. Let $d_{ij}$ be the distance between the bins *i* in *K* and *j* in *L*. In the formal setting of WD, this distance could be measured in units of length between actual piles of earth. Subsequently, the WD is also specified in units of length by default. But more generally, $d_{ij}$ could be set as the difference between probability measures in the corresponding bins *i* and *j*, and hence need not be measured in units of length (*vide infra*). We consider all possible transformations of *K* into *L*. We represent such a transformation by the matrix of values $\left[f_{ij}\right]$ with $f_{ij}$ denoting the mass, (i.e. probability) transferred from bin *i* in *K* to bin *j* in *L*. The WD between *K* and *L* is given by the optimal objective function value of the following optimization problem.

$$\min\;\sum_{i=1}^{k}\sum_{j=1}^{l}d_{ij}f_{ij},$$ (7)

$$\sum_{j=1}^{l}f_{ij}\leq K\left(i\right),i=1,\ldots ,k,$$ (8)

$$\sum_{i=1}^{k}f_{ij}\leq L\left(j\right),j=1,\ldots ,l,$$ (9)

$$\sum_{i=1}^{k}\sum_{j=1}^{l}f_{ij}=1,$$ (10)

$$f_{ij}\geq 0,i=1,\ldots ,k,\;j=1,\ldots ,l.$$ (11)

Constraints 8) and 9) specify that we cannot transport more probability out of a bin than what is available. Equation 10 ensures we transform all of *K* into *L*. The Wasserstein distance can be computed efficiently by solving this optimization problem as a transportation problem (Ahuja et al., 1993).

We illustrate the Wasserstein distance computation on two example Betti sequence probability distributions in Figure 2. The two Betti sequences are presented in the top row of Figure 2 as the number of intersections with the $\beta_{1}$ barcode as a function of the radius of the balls (used to construct the VR complex). The middle row of Figure 2 presents the corresponding Betti sequence probability distributions. Recall that we normalize the Betti sequence to create the *Betti sequence probability distribution* indexed by the number of intersections. Each such distribution adds up to a total probability of 1, by definition. We then compute the Wasserstein distance to transform the probability *K* on the left to probability *L* on the right. The third row of Figure 2 illustrates the optimal way to redistribute the area associated with the probability distributions to make the two equal. This transformation is effected by first moving the $f_{01}=0.1$ probability from intersection 0 to intersection 1 (the red box). This move contributes 0.1×|0−1|=0.1 to the overall distance. In the next step, the $f_{32}=0.2$ probability is moved from intersection 3 to intersection 2 (the cyan box). This move contributes 0.2×|2−3|=0.2 to the total distance. Finally, we move the $f_{31}=0.1$ probability at intersection 3 to intersection 1 (the green box), which contributes 0.1×|1−3|=0.2 to the distance. Hence the Wasserstein distance between the two Betti sequences is given as 0.1+0.2+0.2=0.5.

#### 2.2.3 Application to Ring Polymers

To study the dynamic fluctuations to ring polymer shape, the $\beta_{1}$ barcode for each ring polymer atom representation is determined at time *t* and the compared to at time t+1. In our application to ring polymer shape comparison, we set $k=l=B_{1}$. This corresponds to the largest value observed in any Betti sequence in the entire data set, i.e., the largest number of bars in the $\beta_{1}$ barcode of any ring polymer (at any radius value). The Wasserstein distance between time sequential Betti sequence probability distributions is then determined. For a trajectory with *N* snapshots, we compute the Wasserstein distance vector with N−1 entries, with the *t*th entry specifying the Wasserstein distance between snapshots *t* and t+1. The value $d_{ij}$ is set to |i−j|.

$$WD=\left[\begin{array}{cccc} wd_{1,2} & wd_{2,3} & \cdots & wd_{N-1,N} \end{array}\right].$$ (12)

Finally, in recognizing that the Wasserstein distance vector WD captures the fluctuation in shape over time by measuring distances between adjacent snapshots, we performed a Fourier transform of WD, followed by principal component analysis. We then used the coefficients for the two largest frequencies for comparing the characteristic fluctuations in ring polymer shape across different chemical systems.

We have made the Python code and sample data available for the main calculations in a GitHub repository (Hu et al., 2020).

## 3 Results and Discussion

### 3.1 System 1: The Kob–Andersen Glass

The RPMD simulation of the Kob–Andersen glass forming system was taken from Markland et al. (2011, 2012) and contained atoms types *A* and *B* with different degrees of atomic delocalization. This was determined by their respective *de Broglie* thermal wavelength $\text{Λ}=\sqrt{\frac{\beta \hbar^{2}}{m}}$, where the mass for particle *A* was a magnitude smaller than particle *B* and consequently particle *A* exhibited more quantum fluctuations than its counterpart. The cubic box consisted of 172 type *A* and 44 type *B* particles for a total of 216 particles. The path integrals were discretized into *p* Trotter slices (beads) with P=64 for both particles, with 3,000 ps (5 x 10^6^ steps with 0.6 fs time steps) of configurations saved every 1,000 steps (0.6 ps) yielding a total of 5,000 snapshots.

#### 3.1.1 Static Atomic Uncertainty Metrics

For the extreme case of the delocalized type *A* and highly localized type *B* atoms in the Kob–Andersen glass, all metrics that evaluate shape are highly differentiated (Supplementary Table S1). In the case of the bead centroid metrics, the radii of gyration are 0.19 and 0.017 Å for the *A* and *B* atoms, respectively. These values belie distributions in the individual distances of the polymer beads from the centroid that are statistically very different and have nominal overlap, as illustrated in Supplementary Figure S1A. Analysis of the R_*g*_ tensors indicates that the *A* atoms are much more aspherical ($b=7.67\cdot 10^{-2}$, Eq. 3) than *B* atoms $\left(b=5.84\cdot 10^{-3}\right)$, and similarly more acylindrical (Supplementary Table S1). Neither atom type is anisotropic, having $\kappa^{2}$ values of $10^{-4}-10^{-5}$ (Eq. 5). The pair-wise bead-bead distance distributions have little overlap for *A* and *B* atoms (Supplementary Figure S1B), which in turn is reflected in the imaginary time mean square displacement for the *A* and *B* atoms as demonstrated in Supplementary Figure S2.

#### 3.1.2 Homology and Persistent Homology Metrics

**FIGURE 3:**
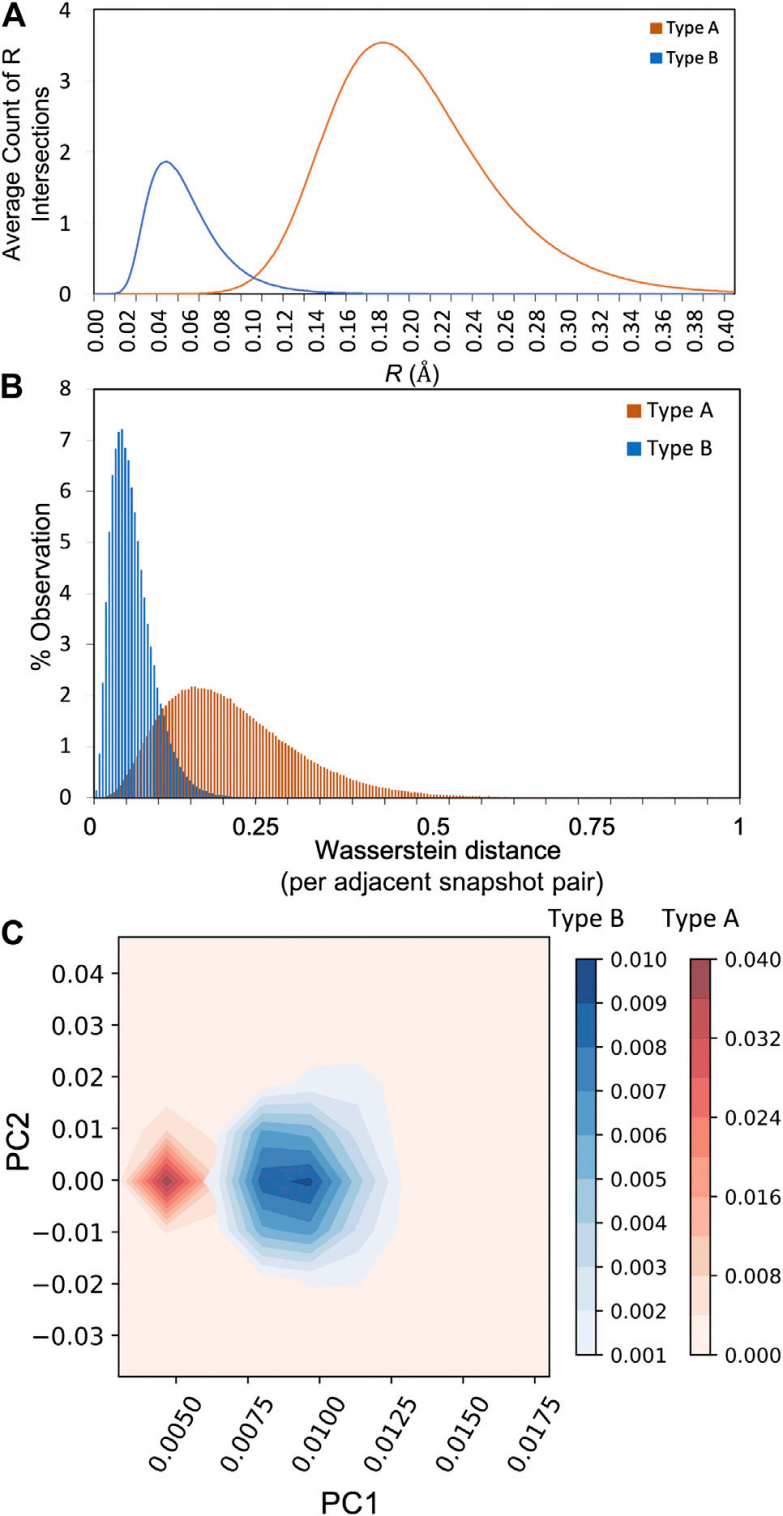
**(A)** The average Betti sequence distribution of all type A and type B atoms **(B)** Distribution of Wasserstein distances between adjacent snapshots in time observed over the entire simulation trajectory **(C)** Principal components analysis capturing 90% of the total variance in the datasets using trajectory windows of ± 20 snapshots (12 ps).

The alternative shape metric based on the Betti sequence of all *A* and *B* atoms is presented in Figure 3. The Betti sequence distribution, which captures the number of intersecting radii as a function of *R*, exhibits two distinctly different distributions in this case. The distribution of Wasserstein distances for the type *A* and *B* atoms are significantly different, where the *A* atoms explore a much broader shape space than *B* (meaning there is more variation in the Betti sequence distribution from one snapshot to the next for type *A*). Further, the magnitude of the Wasserstein distances are much larger for type *A* relative to *B*, meaning that from one snapshot to the next there are large changes to the shapes that the *A* ring polymers adopt. This is further demonstrated by monitoring the time evolution of Wasserstein distances, as shown in Supplementary Figure S3. To quantify the fluctuation in Wasserstein distances, the Fourier transform was studied for type *A* and *B* atoms, followed by principal component analysis. As illustrated in Figure 3C, the first principal components (PC1) for both atom types are clearly well separated, and along with PC2, are able to explain 90% of the variance. The Fourier transform was examined with different lengths of sampling duration, with no appreciable changes observed (Supplementary Figure S4). The Kob–Andersen glass forming system thus represents a proof of principle that a broader suite of shape metrics may be suitable for understanding shapes of ring-polymer representations of atoms, and that persistent homology metrics can reveal identifying characteristics of atoms with dramatically different quantum behavior.

### 3.2 System 2: Aqueous 4 M Hydrochloric Acid

Given the effectiveness of the Wasserstein distance and its Fourier transform in distinguishing atoms in the Kob–Andersen Lennard Jones system, it is thus pertinent to examine the ability of such new methods to reveal varying properties of atoms with much closer nuclear quantum behavior. To test an extreme case, where normal distance-based shape metrics do not indicate significant variations in quantum mechanical behavior, we turn to the identification of proton-transferring H-atoms in a 4 M HCl aqueous solution.

The TRPMD simulation of the 4 M HCl solution was taken from the work of Napoli et al. (2018a). The cubic box of length 14.926 Å consisted of 102 water molecules, eight excess H^+^ and Cl^−^. The path integrals were discretized into P=32 Trotter slices for all atoms. A total of 123 ps of path integral simulations using the revPBE0-D3 hybrid functional (Adamo and Barone, 1999; Grimme et al., 2010; Goerigk and Grimme, 2011) with a 2 fs time step and sampling frequency were analyzed for a total of 61,555 snapshots. Analysis of topological properties of the ring polymers focused on the H-atoms, where they were split into two primary groups based on whether or not they underwent proton transfer during the course of the trajectory. To identify transferring H-atoms, a sequential filtration process was employed. First, the centroids of all O- and H-atoms were examined, wherein zundel cations were identified by employing an O–H distance criterion of 1.3 Å (Napoli et al., 2018; Schran et al., 2018). Within this set, time windows of 40–200 fs were then examined and any changes to the connectivity of the H-atom to different O-atoms were examined. Changes to O-atoms bonded to the H-atom was then identified, (i.e. an H-atom is connected to O_1_, then forms a zundel with the H-atom shared between O_1_ and O_2_, and then the H-atom forms a single bond with O_2_), and subsequently the connectivity of each bead of the H-atom ring polymer was analyzed to understand the timescale associated with all 32 beads changing O-atom partners. An average time of 40 fs was observed for the complete proton transfer (PT) of all 32 beads. Using this criterion (that all 32 beads must change O-atom partners) within a 40 fs time window (+ 20 fs relative to the center of the time window) a total of 2,283 proton transfer events were identified during the simulation trajectory. Non-PT H-atoms were identified as those wherein the 32 beads did not change their covalent connectivity during a 40 fs time window. In total, there are 1,267 windows of time where H-atoms do not undergo *p*T. This yields a total of 98,638 snapshots and is comparable to the 2,283 × 40 = 91,320 snapshots in the proton-transferring dataset.

#### 3.2.1 Shape Metrics

Comparison of the R_*g*_ of H-atoms undergoing PT and those chemically inert H-atoms yields nearly identical values of 0.1211 Å and 0.1206 Å, respectively (Supplementary Table S3). This is in good agreement with prior observations of nearly identical R_*g*_ in other room-temperature proton transferring H-atoms in formic acid (Ivanov et al., 2015). Similarly there is little discrimination in shape factors, with the shape anisotropy only being slightly larger for proton transferring atoms ($\kappa^{2}$ of $1.36\cdot 10^{-2}$) relative to non-transferring counterparts ($\kappa^{2}$ of $8.03\cdot 10^{-3}$; Supplementary Table S2). Analysis of the underlying distribution of distances between all beads and the centroid reveals significant overlap (Supplementary Figure S5), and indeed, applications of Student’s t-test reveal the centroid distance distributions to be statistically equivalent (Supplementary Table S3).

Interestingly, a slightly better identification of PT and non-PT H-atoms is obtained using the bead–bead pairwise distance distribution, which passes the student t-test, however, the average values are still nearly identical, at 0.2155 Å and 0.2151 Å. A more clear delineation is further obtained in the distributions of the Betti sequences of the H-atoms undergoing proton transfer relative to the unreactive atoms (Supplementary Figure S5), where the average distances for reactive and unreactive atoms is 0.1286 vs. 0.1263, respectively, also being statistically significant and passing Student’s t-test (Supplementary Table S3). This suggests that the Betti sequence distribution, which contains more information about the ring-polymer shape, is a more sensitive shape metric than the methods based on distances within the ring polymer.

#### 3.2.2 Persistent Homology Metrics

**FIGURE 4:**
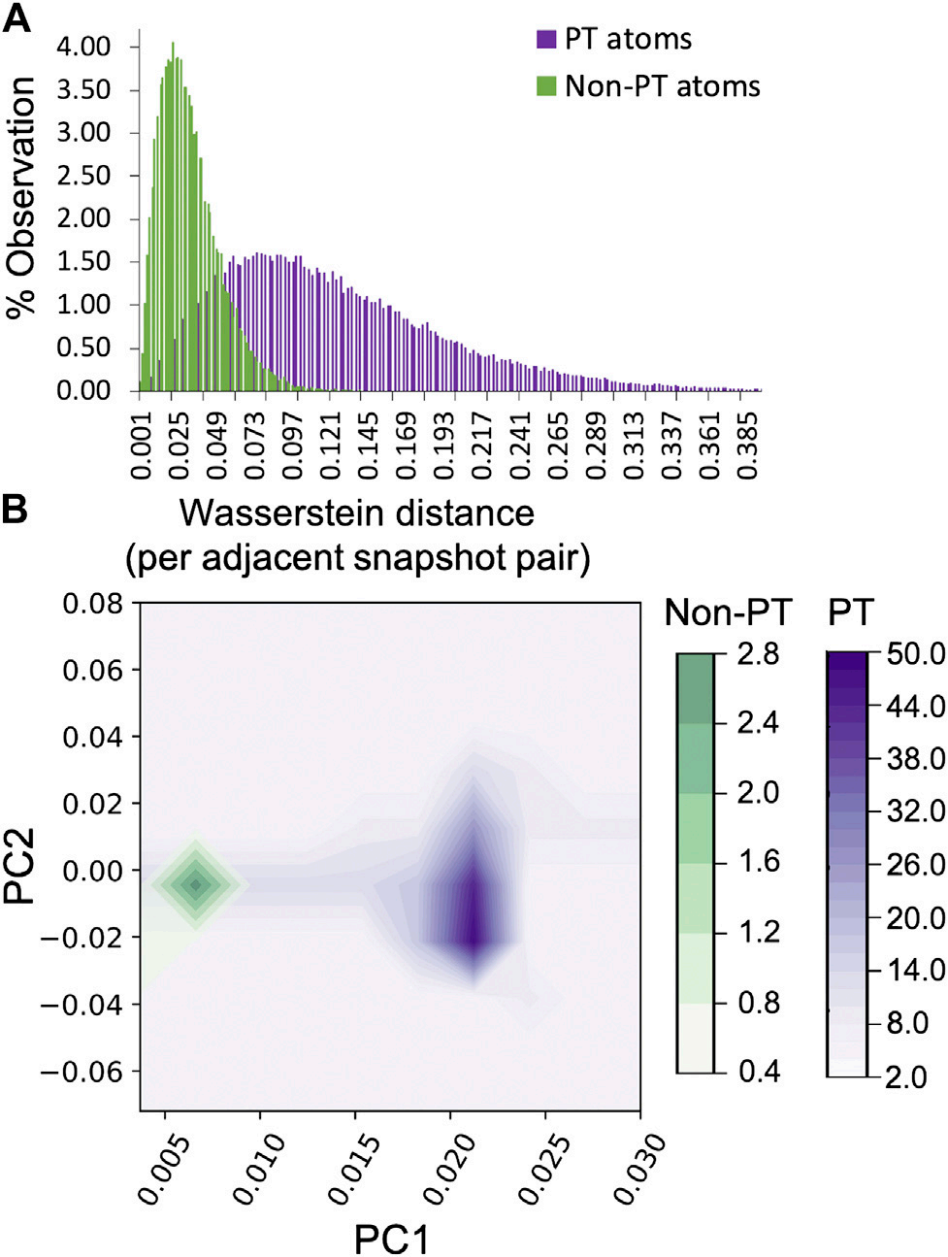
Analysis of the Wasserstein distance characteristics of adjacent *t* and t+1 snapshots for proton transferring (PT) and non-PT H-atom ring polymers **(A)** The distribution of Wasserstein distances observed over all PT and non-PT ring polymers **(B)** Principle component analysis of the PT and non-PT H-atoms capturing 90% of the total variance in the datasets.

As in the Kob–Andersen glass, the Wasserstein distances between PT and non-PT H-atoms in adjacent frames were then examined to identify the fluctuation in shape from one snapshot in the trajectory to another. Unlike the ensemble shape distributions for these atoms, the distribution of the Wasserstein distances for the two sets of H-atoms *is very well-separated* (Figure 4A). The PT atoms exhibit a larger range of accessible shapes, (i.e. the distribution broad), and the fluctuation in shape (which leads to a large cost for changing the Betti distribution from one snapshot to the next) is significantly larger than in the case of non-PT atoms, sampled within a similar 40 fs time window. The fluctuations themselves are illustrated in Supplementary Figure S6. This illustrates that the time dependent fluctuations in shape may be an alternative metric for identifying variations in nuclear quantum behavior between atom sets. Perhaps just as important is the observation that the NQEs may manifest themselves differently for the static vs. dynamic features of ring polymers. The Fourier transform of the Wasserstein distances was then performed and the two dominant principal components plotted in Figure 4B. In contrast to the Kob–Andersen glass system, the correlation between the first and second principal components is much higher, however they are still clearly differentiated for the PT vs. non-PT atoms. In combination, these data demonstrate that metrics based upon the fluctuation of atom delocalization in time are highly sensitive to quantum behavior, being able identify such phenomena when traditional ensemble averaged shape metrics based upon distance criterion (like the gyration radius) are inadequate.

## 4 Conclusion

As the pervasiveness of path integral methods increases within the applied computational chemistry community, new tools are needed to identify atoms where NQEs may be relevant and understand the role of NQEs in reactive processes. While a few metrics exist that identify variations in atomic position uncertainty, they are optimal for systems where the difference in uncertainty is large between different atom types. This work expands the set of available tools to study the shape of the delocalization of atomic positions, the uncertainty associated with NQEs, using persistent homology. Further, the chemical information associated with the time evolution of shape has not been investigated previously. Here, we demonstrate that compared to static distributions the time-dependent persistent homology metrics can provide a clearer way to identify atoms where NQEs are important and to distinguish atoms of different kinds or in different chemical environments. Reactive hydrogen atoms during proton transfer exhibit much larger fluctuations in time of their ring polymer shape than non-reactive counterparts. We believe that the utility of metrics that capture the fluctuations of the atom delocalization in time is generalizable to other reactive chemical systems, and in turn that this provides a means to extract information on reactivity from the quantum behavior of the system, a topic that has not received consideration within the literature.

## Data Availability

The raw data supporting the conclusion of this article will be made available by the authors, without undue reservation.
