# Supplementary material for: Persistent Homology Metrics Reveal Quantum Fluctuations and Reactive Atoms in Path Integral Dynamics
Source: Front Chem. 2021 Mar 5;9:624937. doi: 10.3389/fchem.2021.624937 (PMC7973227; doi:10.3389/fchem.2021.624937)
Supplement: Supplementary file 2 [file datasheet2.pdf]

Persistent Homology Metrics Reveal Quantum Fluctuations and Reactive Atoms in Path Integral Dynamics

1 SUPPLEMENTARY TABLES AND FIGURES

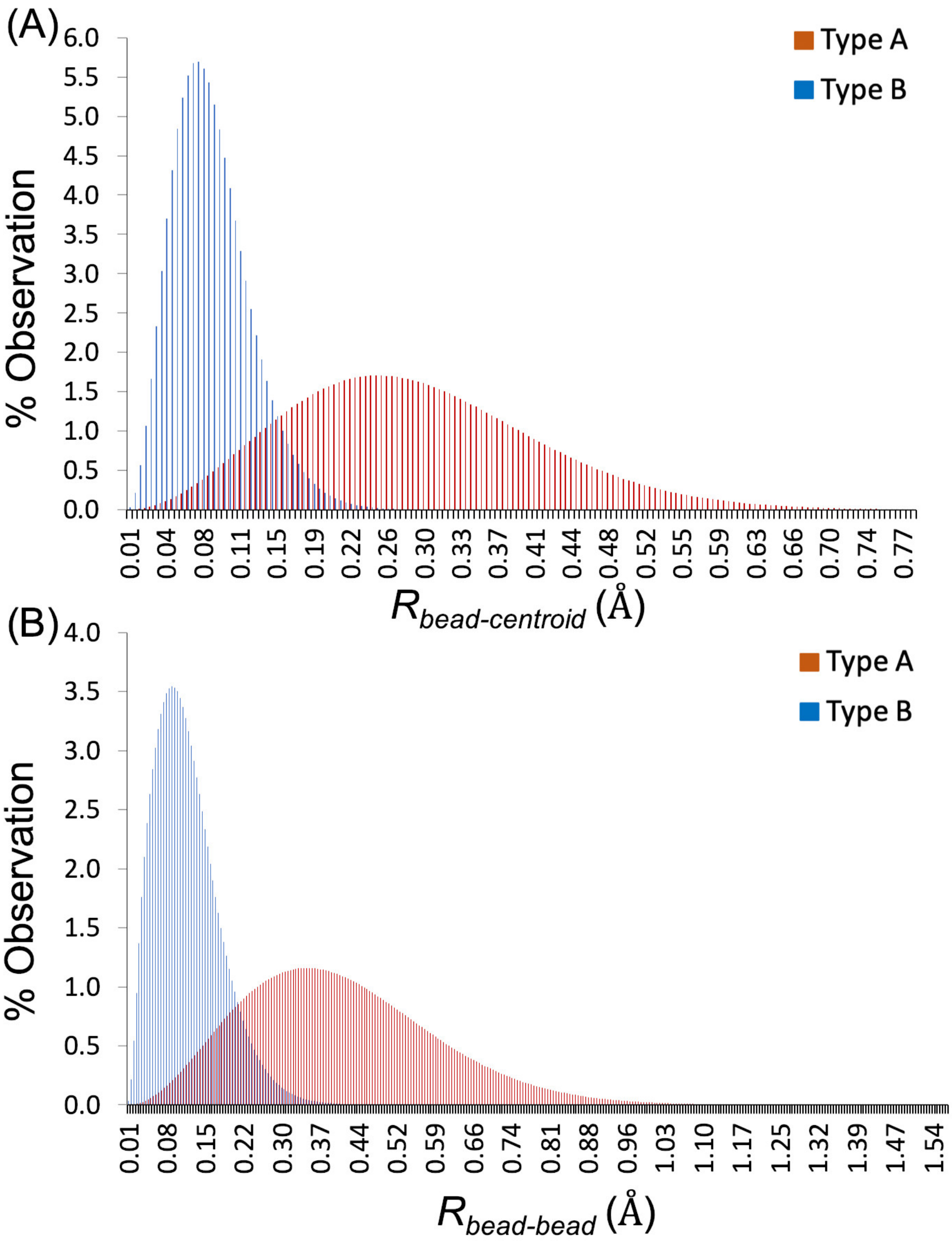

Figure S1: Analysis of shape metrics for the Kob–Anderson glass systems. (A) Distribution of bead to centroid distances for type A and B atoms. (B) Distribution of pairwise distances between all beads within the A and B ring polymer atoms.

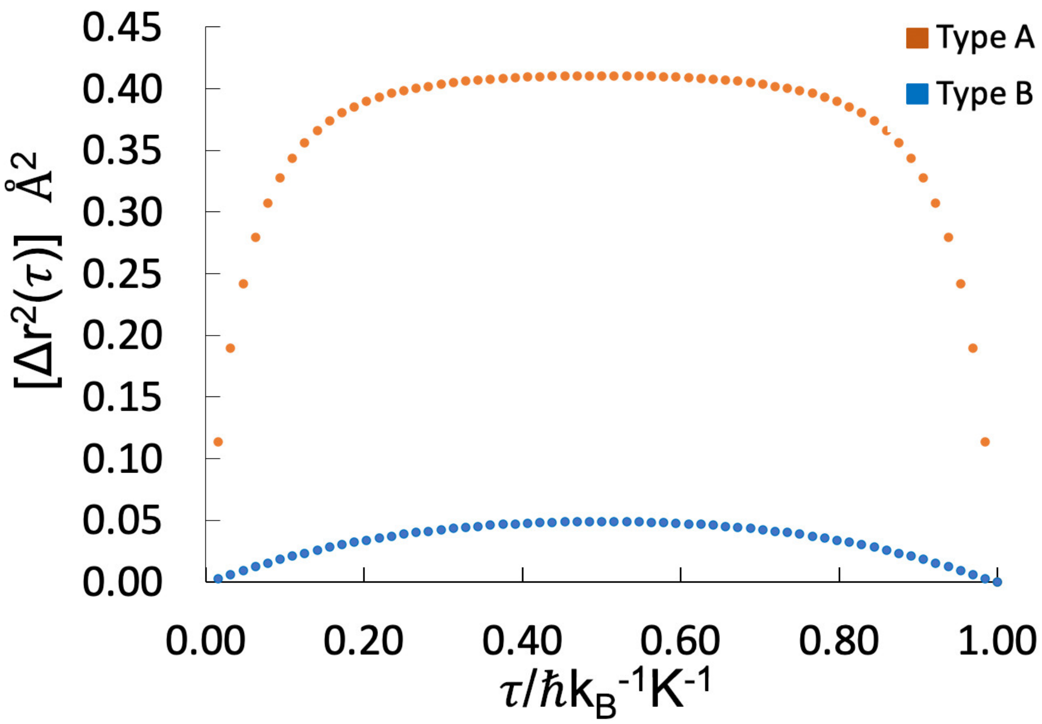

Figure S2: Imaginary mean square displacement of type A and B atoms in the Kob-Anderson glass.

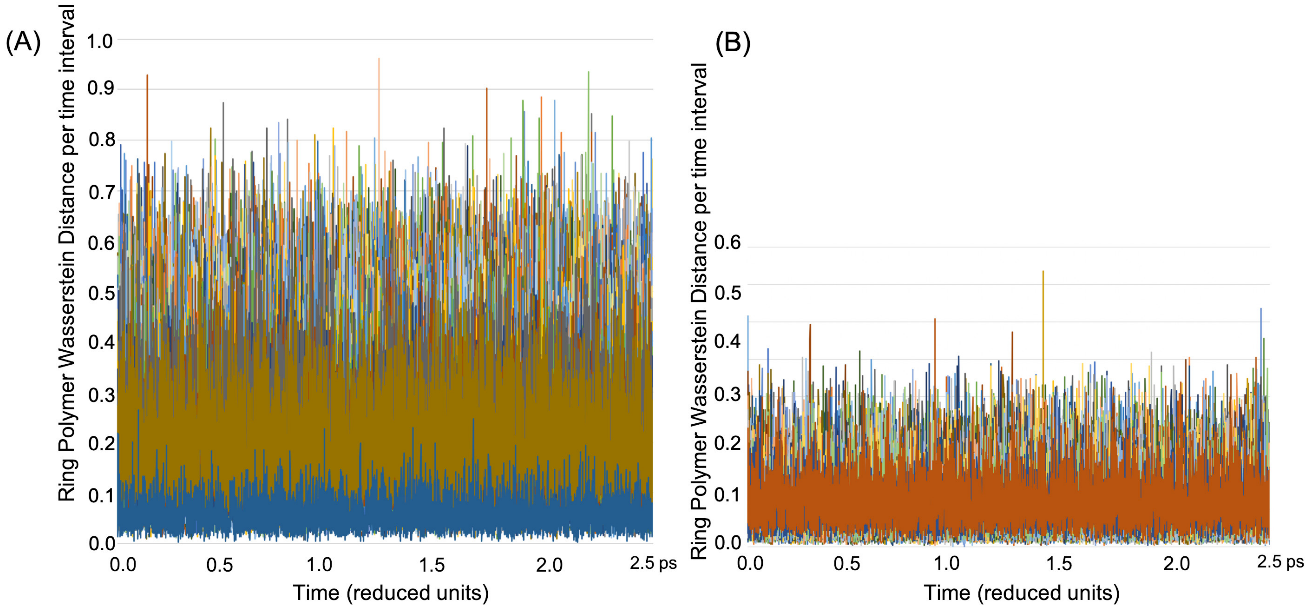

Figure S3: Time evolution of the Wasserstein distances from adjacent snapshots in time for the (A) A and (B) B ring polymer atoms in the Kob–Anderson glass.

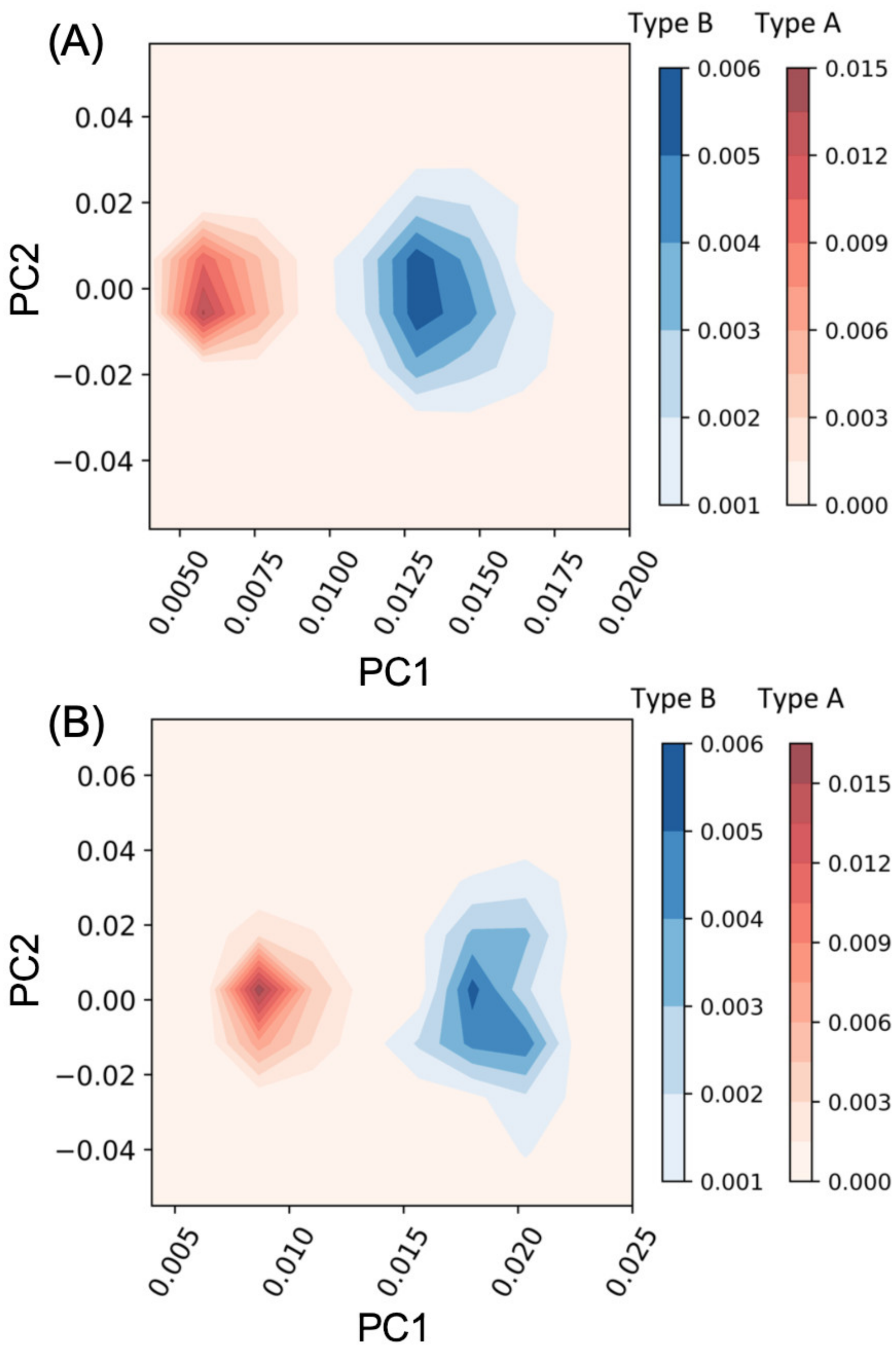

Figure S4: Principal components analysis of the Fourier transform of the Wasserstein distances of the A and B atoms in the Kob–Anderson glass, capturing 90% of the total variance in the datasets using trajectory windows of (A) +/- 40 snapshots (24 ps) and (B) +/- 80 snapshots (96 ps).

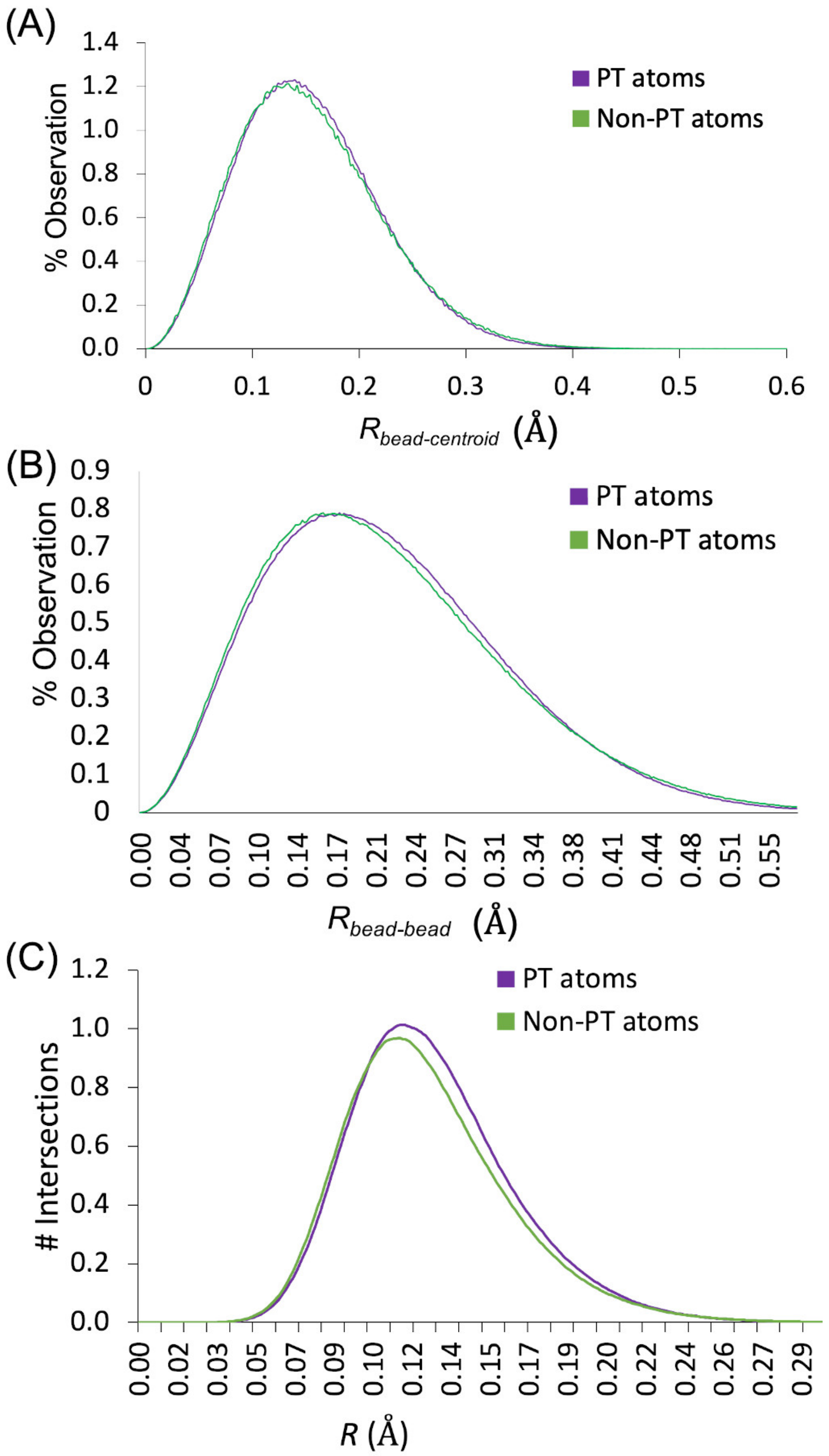

Figure S5: Analysis of the shape metrics of the proton transferring (PT) and non-PT H-atoms in 4M HCl. (A) Distribution of distances between each bead and the centroid for the proton-transferring (PT) and non-PT H-atoms, (B) Distribution of distances between each pair of beads for the PT and non-PT H-atoms, C) Betti sequence distribution of PT and non-PT ring polymers.

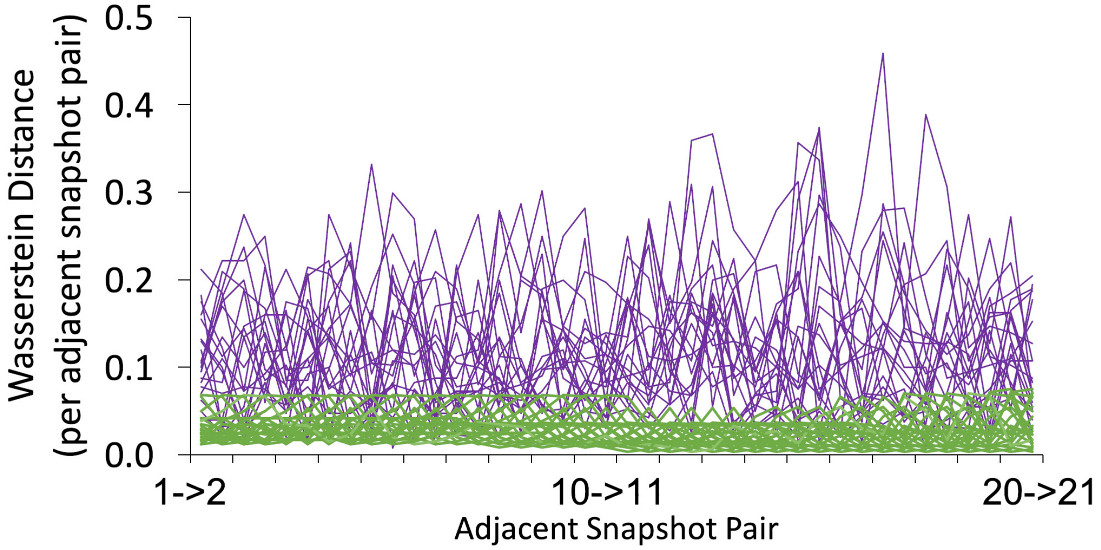

Figure S6: Time evolution of the Wasserstein distances from adjacent snapshots in time for the PT (purple) and non-PT (green) polymer atoms in 4M HCl.

Type A, R_g = 0.19 Å			
	$6.14 \cdot 10^{-2}$	$4.10 \cdot 10^{-4}$	$-9.37 \cdot 10^{-4}$
	$4.10 \cdot 10^{-4}$	$6.39 \cdot 10^{-2}$	$1.54 \cdot 10^{-4}$
	$-9.37 \cdot 10^{-4}$	$1.54 \cdot 10^{-4}$	$6.30 \cdot 10^{-2}$
Type B, R_g = 0.012 Å			
	$3.89 \cdot 10^{-3}$	$-3.01 \cdot 10^{-5}$	$2.08 \cdot 10^{-5}$
	$-3.01 \cdot 10^{-5}$	$3.92 \cdot 10^{-3}$	$-2.08 \cdot 10^{-5}$
	$2.08 \cdot 10^{-5}$	$-2.08 \cdot 10^{-5}$	$3.94 \cdot 10^{-3}$
	Aphericity b	Acylindricity c	Shape Anisotropy κ^2
Type A	$7.67 \cdot 10^{-2}$	$2.50 \cdot 10^{-3}$	$1.35 \cdot 10^{-4}$
Type B	$5.84 \cdot 10^{-3}$	$2.83 \cdot 10^{-5}$	$1.34 \cdot 10^{-5}$

Table S1. Radii of gyration, R_g (Eqn. 1), and diagonalized R_g tensor (in Å) for Type A and B atoms in the Kob–Anderson glass, alongside the respective asphericity (Eqn. 2), acylindricity (Eqn. 3) and shape anisotropy (Eqn. 4).

Non-PT Atoms, R_g = 0.1206 Å			
	$5.52 \cdot 10^{-3}$	$-4.75 \cdot 10^{-4}$	$-6.38 \cdot 10^{-4}$ xxx
	$-4.75 \cdot 10^{-4}$	$4.04 \cdot 10^{-3}$	$-1.02 \cdot 10^{-3}$
	$-6.38 \cot 10^{-4}$	$-1.02 \cdot 10^{-3}$	$5.01 \cdot 10^{-3}$
PT Atoms, R_g = 0.1211 Å			
	$5.79 \cdot 10^{-3}$	$-7.71 \cdot 10^{-4}$	$-1.45 \cdot 10^{-3}$
	$-7.71 \cdot 10^{-4}$	$3.83 \cdot 10^{-3}$	$-1.02 \cdot 10^{-3}$
	$-1.45 \cdot 10^{-3}$	$-1.02 \cdot 10^{-3}$	$5.07 \cdot 10^{-3}$
	Aphericity b	Acylindricity c	Shape Anisotropy κ^2
Non-PT Atoms	$2.29 \cdot 10^{-4}$	$1.48 \cdot 10^{-3}$	$8.03 \cdot 10^{-3}$
PT Atoms	$2.58 \cdot 10^{-4}$	$1.96 \cdot 10^{-3}$	$1.36 \cdot 10^{-2}$

Table S2. Radii of gyration, R_g (Eqn. 1), and diagonalized R_g tensor (in Å) for proton transferring (PT) and non-proton transferring (non-PT) atoms in 4M HCl, alongside the respective asphericity (Eqn. 2), acylindricity (Eqn. 3) and shape anisotropy (Eqn. 4).

**Centroid Dist. Distribution**	PT atoms	Non-PT atoms
R_g (Å)	0.1211	0.1206
Stdev(Å)	0.0649	0.0673
t-value	0.7689	
p-value	0.4419	
t-test	fail	
**Pairwise Dist. Distribution**	PT atoms	Non-PT atoms
$\bar{x}_P$ (Å)	0.2155	0.2151
Stdev(Å)	0.1023	0.1062
t-value	12.6584	
p-value	$1.0052 \cdot 10^{-36}$	
t-test	pass	
**Betti sequence dist.**	PT atoms	Non-PT atoms
$\bar{x}_B$ (Å)	0.1286	0.1263
Std(Å)	0.03537	0.0360
t-value	129.9054	
p-value	$0.0 < 0.01$	
t-test	pass	

Table S3. Shape Metrics and their underlying distributions for H-atoms undergoing proton transfer (PT) and unreactive (non-PT) atoms, including statistical students t-test values.
