# Supplementary material for: Persistent Homology Metrics Reveal Quantum Fluctuations and Reactive Atoms in Path Integral Dynamics
Source: Front Chem. 2021 Mar 5;9:624937. doi: 10.3389/fchem.2021.624937 (PMC7973227; doi:10.3389/fchem.2021.624937)
Supplement: Supplementary file 3 [file table1.doc]

Nov. 1, 2019

YZ

Department of Nuclear, Plasma, and Radiological Engineering

University of Illinois, Urbana-Champagne

Dear Professor YZ;

We are pleased to submit the attached manuscript “*Persistent Homology Metrics Reveal Quantum Fluctuations and Reactive Atoms in Path Integral Dynamics*” to Frontiers in Chemistry, the special issue on “Integrating Timescales from Molecules Up”. This work provides a new set of metrics to quantify quantum delocalization of atoms from path integral molecular dynamics methods. The new metrics are based upon computational topology, specifically persistent homology (PH), as a means to characterize the shape of atoms described by ring polymers. In particular, we demonstrate that PH-based metrics are more sensitive to quantum delocalization than traditional metrics, like the gyration radius, that are based upon pair-wise distances. By studying the time-evolution of the ring polymer shapes, we further show that the fluctuations of shape are much more sensitive to quantum phenomena than the ensemble averaged shape metrics. These new metrics are demonstrated for two chemical systems that have systematically different quantum behaviors of the particles therein.

As the pervasiveness of path integral methods increases within the applied computational chemistry community, new tools are needed to identify atoms where NQEs may be relevant and understand the role of NQEs in reactive processes. We believe that the utility of metrics that capture the fluctuations of the atom delocalization in time is generalizable to other reactive chemical systems, and in turn that this provides a means to extract information on reactivity from the quantum behavior of the system, a topic that has not received consideration within the literature.

Based upon their experience in computational chemistry, path integral dynamics, and topology, we suggest the following as suitable reviewers:

Theresa Head-Gordon

College of Chemistry

University of California, Berkeley

Email: [thg@berkeley.edu](mailto:thg@berkeley.edu)

Pierre-Nicholas Roy

Department of Chemistry

University of Waterloo

Email: [pnroy@uwaterloo.ca](mailto:pnroy@uwaterloo.ca)

Lori Ziegelmeier

Department of Mathematics

Macalester College

Email: lziegel1@macalester.edu

We look forward to your editorial consideration;

Aurora Clark

Professor of Chemistry

Fellow, ACA and AAAS

Director, Center for Institutional Research Computing

Washington State University

Pullman, WA 99164

Email: [auclark@wsu.edu](mailto:auclark@wsu.edu)
